# Rational design of glycosaminoglycan binding cyclic peptides using cPEPmatch

**DOI:** 10.1016/j.csbj.2024.07.016

**Published:** 2024-07-20

**Authors:** Brianda L. Santini, Margrethe Gaardløs, Dariusz Wyrzykowski, Sven Rothemund, Anja Penk, Martin Zacharias, Sergey A. Samsonov

**Affiliations:** a Center for Functional Protein Assemblies, Technical University of Munich, Ernst-Otto-Fischer-Straße 8, Garching, Germany; b Department of Chemistry, University of Gdańsk, Poland; c Unit Peptide Technologies, Liebigstraße 21, Leipzig, Germany; d Institute of Medical Physics and Biophysics, Härtelstr. 16/18, Leipzig, Germany

**Keywords:** Cyclic peptides, Glycosaminoglycans, Antithrombin, Rational design, Molecular dynamics, Nuclear magnetic resonance, Isothermal titration calorimetry

## Abstract

Cyclic peptides present a robust platform for drug design, offering high specificity and stability due to their conformationally constrained structures. In this study, we introduce an updated version of the Cyclic Peptide Matching program (cPEPmatch) tailored for the identification of cyclic peptides capable of mimicking protein-glycosaminoglycan (GAG) binding sites. We focused on engineering cyclic peptides to replicate the GAG-binding affinity of antithrombin III (ATIII), a protein that plays a crucial role in modulating anticoagulation through interaction with the GAG heparin. By integrating computational and experimental methods, we successfully identified a cyclic peptide binder with promising potential for future optimization. MD simulations and MM-GBSA calculations were used to assess binding efficacy, supplemented by umbrella sampling to approximate free energy landscapes. The binding specificity was further validated through NMR and ITC experiments. Our findings demonstrate that the computationally designed cyclic peptides effectively target GAGs, suggesting their potential as novel therapeutic agents. This study advances our understanding of peptide-GAG interactions and lays the groundwork for future development of cyclic peptide-based therapeutics.

## Graphical Abstract

**Figure:**
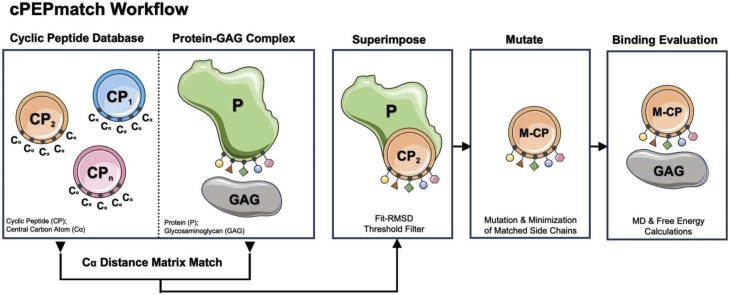

## 1 Introduction

Cyclic peptides are an emerging class of compounds with unique structural and chemical properties that make them promising candidates for drug design [41]. In contrast to linear peptides, cyclic peptides have a more rigid and constrained structure that underlies their improved stability against proteases and, therefore, potentially higher bioactivity [13], [23]. A peptide can be made cyclic by forming a covalent bond using various chemical modifications, including disulfide bridging, amide bond, sidechain to sidechain covalent binding, staples, or combining any of the above [3]. As a result, cyclic peptides can adopt various well-defined secondary structures, such as alpha-helices, beta-sheets, and turns – which confers their ability to mimic a variety of biological molecules, such as enzymes, antibodies, and hormones, and can be used to modulate or inhibit a range of biological activities, making them a versatile class of therapeutics (London et al., 2010).

Cyclic peptides possess properties that render them particularly attractive as therapeutics, including:

### 1.1 High target selectivity

Cyclic peptides can be meticulously designed to target specific proteins or receptors, facilitating strong and specific interactions with their binding partners. This high degree of selectivity reduces the risk of off-target effects compared to small molecules [36], [6].

### 1.2 Improved stability

Cyclic peptides are more resistant to degradation by proteases and other enzymes in the body compared to linear peptides, making them longer-lasting in the bloodstream and more therapeutically effective [17].

### 1.3 Enhanced pharmacokinetic properties

The compact structure of cyclic peptides contributes to improved pharmacokinetic properties such as increased bioavailability, better permeability across biological barriers, and more effective distribution throughout the body compared to biologics [23]. However, despite these advantages, cyclic peptides still face some limitations in oral availability and cell permeability. Efforts, such as cell-penetrating peptides, stabilization with hydrocarbon linkers, and N-methylation, are being investigated by various research groups to address these issues [41].

Computational tools play a significant role in the rational design of cyclic peptides to mimic proteins. Amongst such tools, we developed a Cyclic Peptide Matching program (cPEPmatch), which provides a proof of principle for the identification of cyclic peptides that mimic binders by matching the backbone structure of a protein against a database of template peptides [31], [32]. We demonstrated that the capacity of cyclic peptides to mimic protein binding sites is based on their capability to adopt a specific three-dimensional structure that allows them to interact with the protein binding partner similarly to the protein itself. Furthermore, for large binding sites, the key to mimic them is to identify the specific amino acid residues responsible for the native interactions termed as hot spots. Protein binding hot spots refer to specific regions on the surface of a protein which mostly contribute in terms of free binding energy. These regions are often composed of a small number of amino acid residues, which establish a favorable interaction with the ligand. Identifying protein binding hot spots before matching a cyclic peptide with cPEPmatch is highly beneficial as it can filter the number of matches [1].

Glycosaminoglycans (GAGs) are linear negatively charged periodic polysaccharides that play critical roles in various physiological and pathological processes in the extracellular matrix. Due to their biological functions and their role in the onset of many diseases, GAGs have become attractive targets for developing therapeutic and diagnostic tools [16]. However, the structural complexity and heterogeneity of GAGs pose significant challenges to designing molecules that can selectively target them [28]. Recently, the ability of cyclic peptides to bind glycosaminoglycans (GAGs) has been exploited for various applications, including the development of therapeutics and diagnostic agents One example is cyclic peptides that bind to heparin (HP), a class of GAGs, developed for use as anticoagulants [38].

HP functions as an anticoagulant through its interaction with the serine protease inhibitor antithrombin-III (ATIII). ATIIIs anticoagulant activity is caused by the selective inhibition of procoagulant proteases (mainly thrombin, factor XIa, and factor Xa) in the blood clotting cascade [26]. HP and heparan sulfate (HS) act as ATIII cofactors, and in their presence, the inhibition activity of ATIII towards its target proteases is increased up to 4000 times [25], [5]. Unlike many GAG-protein interactions, which are predominantly mediated by rather non-specific electrostatic interactions, ATIII requires a highly specific GAG sequence, including an uncommon 3-*O* sulfation group, for the effective mediation of its activity [19], [33]. Taking all this into account, engineering cyclic peptides to mimic the GAG-binding affinity of ATIII presents a promising strategy for modulating anticoagulation activity. This approach aims to demonstrate the potential of our algorithm to design peptides that could influence various GAG-modulated processes, including anticoagulation, cell adhesion, and inflammation [35].

In this study, we report an adaptation of the cPEPmatch approach to design cyclic peptides that specifically target GAGs by mimicking the hot spots of GAG-binding proteins. Mimicking these binding interfaces with cyclic peptides is a challenging task, and it requires a deep understanding of the structure and function of the protein binder interface. GAG-binding regions are often characterized by clusters of positively charged amino acid residues, which can establish electrostatic interactions with the negatively charged GAGs. However, while sequentially consecutive motifs are not typically associated with the corresponding hot spot residues, these residues tend to cluster together discontinuously within the binding proteins establishing positive patches on the protein surface. In this work, we present a modified version of the cPEPmatch code that allows for matching cyclic peptides that target sequentially non-consecutive hot spots. Our approach provides a potential avenue for the development of novel GAG-targeted therapeutics that can potentially modulate GAG functions in various biological processes in a specific manner.

## 2 Materials and methods

### 2.1 Structures

In this study, ATIII was modeled based on the crystal structure in which this protein is bound to a heparin mimetic (PDB ID 1E03, [22]). This dimeric structure consists of ATIII in two different conformations, and the active conformation corresponding to chain A was chosen as this has a higher affinity towards the GAG-ligand [22], [40].

The sequence of the GAG used in simulations with cPEPs is based on the pentamer from the ATIII complexed crystal structure (1E03). In this oligosaccharide the glucosamine nitrogens are replaced by oxygens. Instead, in the study we used this structure with the natural N-sulfation corresponding to.

D-GlcNS6S-α(1,4)-D-GlcA-β(1,4)-D-GlcNS3,6S-α(1,4)-L-IdoA2S-α(1,4)-D-GlcNS6S.

where GlcA denotes glucuronic acid and IdoA denotes iduronic acid, GlcNS N-sulfated glucosamine and additional sulfate groups are marked by their position in the respective sugar chain. This ligand is termed as ligand 1 in the manuscript. In addition, the classical HP sequence D-GlcNS6S-α(1,4)-L-IdoA2S-D-GlcNS6S-α(1,4)-L-IdoA2S-α(1,4)-D-GlcNS6S was modeled and termed as ligand 2 throughout the manuscript. Ligand 2 is not a highly selective ligand for ATIII and was used in our study as a control.

### 2.2 Cyclic peptide matching

The original cPEPmatch workflow consists of four main steps: (1) Cyclic peptide database construction. (2) Identification and characterization of the protein interface to mimic. (3) Interface backbone matching against the cyclic peptide database and conducting structure superimposition. (4) Mutation of the matched amino acids to the side chains corresponding to the protein to mimic. All matches are then refined and evaluated using Molecular Dynamics (MD) simulations and Molecular Mechanics Generalized Born Surface Area (MM-GBSA) [37] free energy calculations, respectively. The original cPEPmatch workflow underwent several modifications as part of this study, which include:

• Migrating from Fortran to Python to improve ease of use, readability, and maintainability.
• In the first and second steps, we characterized the backbone structure of cyclic peptides and protein to mimic, respectively, by calculating distances between backbone atoms of four consecutive residues – based on the notion that the backbone structure of these motifs plays a crucial role in binding interactions [15]. In the GAG binding protein, hot spot residues are in general not present as sequentially consecutive motifs. Thus, we have added a modification for the search for non-sequential motifs.
• In step two, the user can now specify which particular amino acids to target, enhancing the selection process for the interface mimicry.
• In step three, a range of motif lengths from four to seven amino acids can be selected for matching.
• In step four, the matched side chains are replaced and minimized using Modeller [29] to reduce steric clashes and optimize the overall stability of the structure.

The source code for cPEPmatch and the associated cyclic peptide database are available for public access in the GitHub repository at https://github.com/briandasantini/cPEPmatch. This resource provides comprehensive insights into the software's functionality and usage guidelines. Additionally, the database includes detailed information on each entry, such as PDB ID, cyclization type, peptide length, secondary structure, and other relevant features, along with the cleaned PDB files with the cyclic peptide structures.

In our ATIII cyclic peptide matching study, we fine-tuned the search parameters to identify the most suitable matches for this complex system. A ‘good match’ was determined through visual inspection using VMD [11], focusing on selecting cyclic peptides that ensure correctly positioned matched side chains, thereby avoiding interference with other ATIII strands. The optimal results were achieved with a motif size of 5 residues; ideally, larger motifs would capture more interactions. However, those with 6 residues failed to yield any viable matches at our established Fit-RMSD threshold of 3 Å. This threshold was chosen because it allows for the flexibility of key side chains like R and K, providing displacement tolerance without compromising the structural integrity or resemblance to ATIII. The Fit-RMSD threshold is pivotal as it dictates how closely the cyclic peptide’s backbone must align with the targeted protein interface residues to ensure an optimal fit. In cPEPmatch, the hotspot entries for ATIII were defined as residues contributing less than −5 kcal/mol to the binding energy, according to MM-GBSA calculations. These include nine residues: K101, R34, R8, K112, N32, K6, R116, R33, T31, listed in descending order of their favorable free energy contributions [30].

cPEPmatch’s capability to process non-consecutive amino acid motifs enhances and widens its utility for cyclic peptide design, though it increases computational demand due to the exponential growth in possible amino acid combinations. Performance testing with a motif size of 5 and all other matching parameters as reported above on a Linux system with an x86_64 processor, 12 CPU cores, and 16 GB of RAM demonstrated that searches for consecutive motifs are swiftly processed in under a second, while non-consecutive matching took approximately 22 min for all nine reported hotspots. In contrast, our searches involving 5–7 hotspots take between 30 s and 6 min. Thus, when dealing with a large number of hotspots, we recommend segmenting hotspot searches into manageable groups of 5–7 amino acids that are structurally relevant to the wanted interface to mimic, reducing the processing time without sacrificing the relevancy of the analysis.

Following the cyclic peptide matching, MD simulations and free energy calculations were conducted to evaluate the binding of the generated cyclic peptides with both analyzed ligands 1 and 2. Subsequently, an optimization phase was carried out for these cyclic peptides, involving a series of strategic mutations to enhance their binding. This refinement step was followed by additional MD simulations to assess the impacts of these modifications on the peptide-ATIII interactions.

### 2.3 MD simulations

In this step, up to four distinct versions of each cyclic peptide identified as a match were subjected to MD simulations in complex with ligand 1. For all three chosen cyclic peptide matches, we initiated the simulations with the configuration as suggested by cPEPmatch. This initial configuration (version 1) incorporated up to 5 residues mutated based on the matching results. Subsequently, informed by the outcomes from these initial simulations, we introduced alternative mutations in two of the peptides, corresponding to 2JRW and 2LWT structures, to further explore their binding efficiencies. Additionally, for these peptides, simulations were also performed using their wild-type sequences (referred to as version 0) to serve as a comparative baseline. The detailed configurations of the various versions, including the specific mutations and their corresponding sequence variations, are listed in Table 1.

**Table 1:** cPEPs investigated in this study. AA denotes amino acid residues, V. denotes version. Version 0 is the wild-type sequence. Underlined residues are the ones that mimic the residues in the binding site of ATIII. Introduced cysteines for disulfide bridges are in italics. Crosses (x) indicate the corresponding hotspots of the introduced mutations.

ID	AA	V.	Sequence	K6	R8	T31	N32	R33	R34	K101	K112	R116
5EOC	13	0	CQLINTNGSWHIC									
		1	CNRRNTKGSWHIC				x	x	x	x		
2JRW	23	0	CAEPMTLPENYFSERPYHPPPPC									
		1	CAEPMTLPEKYFSENRRHPPKPC	x			x	x	x	x		
		2	CAEPRTLPEKYFKTNRRHPPKPC	x	x	x	x	x	x	x	x	
		3	CARPKTLPKR*C*FSENR*C*RPKPPC	x	x		x	x	x	x	x	x
2LWT	18	0	GRYRRCIPGMFRAYCYMD					x	x			
		1	GTNRRCKPGMFRAYCYMD			x	x	x	x	x		
		2	GTNARCKPGMFRAKCKMR	x		x	x		x	x	x	x
		3	GTNARCRPGKFRAKCKMR	x		x	x	x	x	x	x	x

Note: Version 3 of 2JRW is structurally reinforced with an internal disulfide bridge introduced by mutating two residues to cysteine, enhancing peptide stability.

100 ns MD simulations were performed for all complexes with the cyclic peptides and the pentameric ligand HS, in addition to ATIII and 2JRW version 2 with both ligands 1 and 2. For the cyclic peptide complexes, the simulations were repeated up to three times. The simulations were performed in AMBER20 with ff14SBonlysc force field for protein and GLYCAM06 for GAG parts of the system [18], [24], [4]. To neutralize the system, sodium ions were added, and the systems were solvated with a 15 Å truncated octahedron box using TIP3P water [14]. Energy minimization was performed in two steps: 500 steepest descent cycles with 10^3^ conjugate gradient cycles and 100 kcal/mol/Å^2^ harmonic force restraints on solutes, followed by 3 × 10^3^ steepest descent cycles and 3 × 10^3^ conjugate gradient cycles without restraints. Subsequent heating to 300 K was performed for 10 ps using the same restraints before equilibration for 500 ps at 300 K and 10^5^ Pa in isothermic isobaric ensemble. The production runs were performed at the same conditions as equilibration. Trajectories were evaluated with cpptraj module of AMBER20 and visualized in VMD.

### 2.4 MM-GBSA free energy calculations

Molecular mechanics-generalized Born surface area (MM-GBSA) [27] in AMBER 20 with mode igb = 2 was performed over the MD-trajectories to assess the affinities of the complexes. Per-residue decomposition allowed for a detailed investigation of the contribution of interacting residues.

### 2.5 Umbrella sampling MD

The binding affinities of the analyzed complexes were also determined using the umbrella sampling (US) method, as described by Torrie and Valleau [34]. This involved simulating the ligand's dissociation by modulating the distance between a backbone nitrogen atom in the protein and the ring oxygen of the central sugar residue. We employed a harmonic restraint with a force constant of 4 kcal/mol/Å². The backbone nitrogen atoms from the following residues were utilized for each complex: V44 in ATIII, W10 in 5EOC, Y14 in both versions of 2LWT, and P4 in both versions of 2JRW. This distance was incrementally increased by 1 Å across successive simulation windows, resulting in a total of 30 windows for the cyclic peptides and 40 for ATIII. Each window included an equilibration phase of 0.1 ns followed by a production simulation lasting 10 ns. The distance probability distributions in each window were visually inspected to confirm their overlap. Potential of mean force (PMF) plots were then derived from these distributions using the weighted histogram analysis method (WHAM), utilizing Grossfield's WHAM software [20], [8]. The tolerance for force convergence was set at 0.001 kcal/mol/Å, and a Monte Carlo bootstrap error analysis was conducted with 1000 simulated data sets.

### 2.6 Peptide synthesis

Solid-phase peptide synthesis of the cyclic peptide 2LWT V2 was performed on an automated peptide synthesizer, MultiPep from Intavis AG (Köln, Germany), using standard Fmoc chemistry. The linear peptide was synthesized on arginine(Pbf)-preloaded 2-Cl-trityl resin. We have used Fmoc-L-Arg(Pbf)−2CT resin from Iris Biotech GmbH, Germany with cross linking of 1 % divenylbenzene, loading of 0.56 mmol/g, and mesh size of 100–200. Single couplings were executed with a 4-fold excess of HCTU and Fmoc amino acids in the presence of an 8-fold excess of NMM for 1 h. The fully protected peptide was cleaved from the solid support using a mixture of acetic acid, trifluoroethanol, and dichloromethane (20:20:60, vol%) for 2 h. A rotary evaporator under low vacuum was used to remove the cleavage mixture, followed by five subsequent n-hexane evaporations. The solid, fully protected peptide was obtained after lyophilization from an acetonitrile-water peptide solution. Head-to-tail coupling was performed without prior purification in DMF at a concentration of 1 mg/mL in the presence of 2 equivalents of PyBOP, 2 equivalents of HOBT, and 4 equivalents of DIEA for 2 days. After the head-to-tail coupling was completed, the peptide was separated from DMF by vacuum evaporation and lyophilization, as described above. The side-chain-deprotected cyclic peptide was obtained by applying a mixture of TFA, water, and thioanisole (95:2.5:2.5, vol%) and ether precipitation. The cyclic peptide was purified to a purity greater than 95 % using preparative RP-HPLC (Gilson GmbH, Germany) equipped with a PLRP-S column (300 × 25 mm, Agilent, Germany). The purified and lyophilized head-to-tail-cycled peptides were then oxidized in 0.1 M ammonium bicarbonate at a peptide concentration of 0.1 mg/mL for a duration of 24 h. The formation of the second cycle between two cysteines was monitored by analytical HPLC (Thermo Fisher Scientific GmbH, Ultimate 3000, Germany) and MALDI-MS (Bruker Microflex LT, Germany), which confirmed the expected [M + H]+ mass peak. The final intramolecular disulfide-linked and head-to-tail-cycled peptides were obtained after a second purification step by preparative HPLC. HPLC Conditions: For both analytical and preparative runs, the mobile phases were water (A) and acetonitrile (B), each containing 0.1 % TFA. Samples were eluted with a linear gradient from 5 % B to 90 % B in 30 min for analytical runs and from 10 % B to 60 % B in 90 min for preparative runs.

### 2.7 Solution NMR

Fondaparinux (D-GlcNS6S-α(1,4)-D-GlcA-β(1,4)-D-GlcNS3,6S-α(1,4)-L-IdoA2S-α(1,4)-D-GlcNS6S-OMe) was purchased from Merck (SML1240). Fondaparinux and ligand 1 are different only by a terminal methyl-group that is not participating in the interaction with the ATIII in the corresponding experimental structure. The peptide 2LWT V2 with a head-to-tail cycle and a disulfide bridge between the two cysteines (GTNARCKPGMFRAKCKMR) was used without further purification.

For partial assignment, a 1.5 mM solution of the cyclic peptide was prepared. It contained 20 mM NaPi, 150 mM NaCl, pH = 6.4, with 10 vol% D2O and 27 μM 3-(Trimethylsilyl)propionic-2,2,3,3-d₄ acid (TMSP-d_4_) for referencing the chemical shift scale. Natural abundance ^1^H–^13^C HSQC, ^1^H–^13^C-TOCSY-HSQC (60 ms mixing time with DIPSI), 1 H-1 H TOCSY (60 ms mixing time with mlev and excitation sculpting), and ^1^H–^1^H NOESY (200 ms mixing time and Watergate + presaturation) were acquired on a Bruker Avance III 600 MHz spectrometer equipped with a 5 mm TXI probe at 283 K. Afterwards, Fondaparinux stem solution (5 mM) was added to yield an equimolar ratio of both substances (around 1.1 mM), and another ^1^H–^13^C HSQC was acquired. Referencing was performed according to [9]. For processing, Topspin 4.0.6 (Bruker, Rheinstetten, Germany) and POKY [21] were used. The reference values for C_α_ and C_β_ shifts were taken from RefDB [39].

To investigate the binding of the cyclic peptide to Fondaparinux, all substances were dissolved in NMR Buffer (20 mM NaPi, 150 mM NaCl, pH = 7 with 10 % (v/v) D_2_O) to concentrations of 100 μM (cPEP) or 5 mM (Fondaparinux). This was to ensure comparability with previous studies on ATIII [10]. To 500 μl of the cPEP solution (including 28.5 μM TMSP-d4 for referencing), increasing amounts of Fondaparinux solution were added (totaling 30 μl). For each titration step, a 1D ^1^H NMR spectrum with excitation sculpting [12] was acquired at 283 K (512 scans with a relaxation delay of 2 s, 8k points, and a spectral width of 16.2 ppm) using a 5 mm E-free HCN probe on a Bruker NEO 700 MHz spectrometer. Spectra were zero-filled to 16k points, and an exponential line broadening of 2 Hz was applied. For ligand ratios of 1:0 and 1:1, a ^1^H −^1^ H TOCSY (60 ms mixing time with DIPSI) was acquired to identify trends in shifts despite overlapping backbone resonances in the 1D spectra.

### 2.8 Isothermal Titration Calorimetry (ITC)

The ITC experiments were performed using the AutoITC isothermal titration calorimeter (MicroCal Inc.) at 298.15 K. Briefly, the reagents, namely the cyclic peptide 2LWT V2 and Fondaparinux, were dissolved directly into 20 mM NaPi buffer (150 mM NaCl, pH 7.0). cPEP at a concentration of 1 mM was gradually injected (29 injections, injection volume: 10.02 μl with 2 μl for the first injection only, injection duration: 20 s, injection interval: 240 s, stirring speed: 300 r.p.m.) into the reaction cell containing 0.1 mM Fondaparinux solution. A background titration, consisting of the identical titrant solution (1 mM 2LWT V2) but only the buffer solution in the sample cell, was subtracted from each experimental titration to account for the heat of dilution. The standard procedure (CaCl2 - EDTA titration) was performed to validate the apparatus, and the stoichiometry, K, ΔH results were compared with those obtained for the test kit at Malvern Instruments Ltd (Malvern, UK). ITC data were processed with Origin 7 (MicroCal).

## 3 Results and discussion

### 3.1 Computational analysis

#### 3.1.1 Adapting cPEPmatch to complex binding sites

The original proof-of-principle cPEPmatch code was designed to match target protein-protein structures using cyclic peptides by mimicking sequentially consecutive motifs of four residues. It defines the binding interface of a receptor by selecting all residues that fall within a specific distance cutoff from the associated protein. Once the interface is defined, the focus shifts to the receptor residues, facilitating adaptation for comparison with proteins binding non-protein ligands. However, the unique nature of protein-GAG interactions requires modifications to the matching algorithm. Unlike protein-protein complexes, for which cPEPmatch was initially created, featuring flat binding surfaces with hot spot motifs of a few residues, many GAG-binding proteins including ATIII, present binding sites comprising sequentially non-consecutive residues. These residues are not only distant in sequence but also extend spatially in three dimensions to accommodate elongated GAG molecules. Defining a binding hot spot in these complexes often involves more than four residues, as shown in the analysis of existing structures of protein-GAG complexes in the PDB [30].

**Fig. 1:**
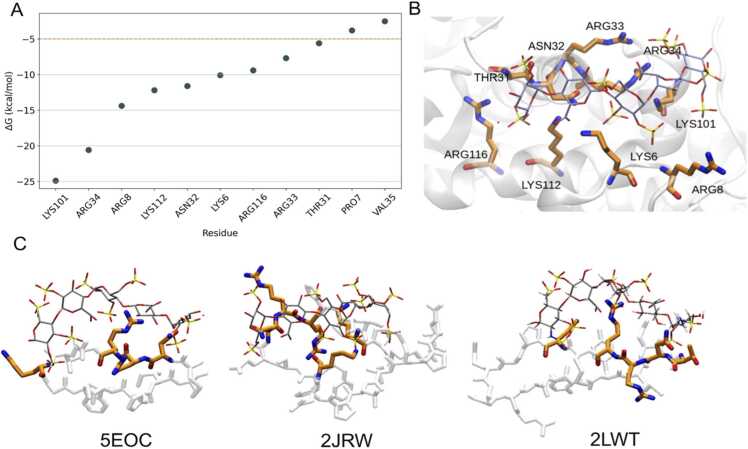
A) Per-residue decomposition of MM-GBSA binding energy for ATIII-ligand 1 complex, highlighting residues with binding energy stronger than –5 kcal/mol. Residues with the values below the dotted orange line are, therefore, included in panel B. B) Visualization of the ATIII binding site (PDB ID 1E03). The protein structure is displayed in a white cartoon representation. The key GAG-binding residues are depicted with carbon atoms in thick orange sticks, while the GAG molecule is represented with carbon atoms in thin gray sticks. C) Three non-consecutive cPEPs matching the ATIII binding site, with the residues corresponding to those from ATIII depicted with carbon atoms in thick orange sticks.

This is exemplified by the ATIII binding site with the HP mimetic as seen in the crystal structure (PDB ID 1E03, [22], Fig. 1B). MD simulations performed for this complex, along with decomposed energies for hot spot residues from MM-GBSA energy calculations, are depicted in Fig. 1A. The binding site, illustrated in Fig. 1B, highlights nine residues with free binding energies below –5 kcal/mol. Notably, these hot spot residues are primarily non-consecutive, except for residues N32-V35 (Fig. 1B). Displayed in the binding groove, these residues encircle the ligand (Fig. 1B), with consecutive residues lining only one side of the circle. To capture the complete representation of the binding groove, it was necessary to consider non-consecutive residues. Therefore, cPEPmatch was updated to allow the matching motif to be defined by non-consecutive residues. We restricted our search to the residues identified to be involved in binding to avoid an excessively large number of non-consecutive combinations within the binding site to exclude residues not participating in the interaction. The code now permits the manual definition of a binding site, including any number of desired residues for each complex. We utilized the nine hot spot residues identified from MM-GBSA calculations to represent the binding site. There remains an option to match all residues within the distance cutoff, beneficial when the binding site is small or when the hot spot residues are not clearly identified. The sum of the binding free energies of all matching residues in the cyclic peptides was calculated and used for ranking the resulting cyclic peptides.

Utilizing the updated cPEPmatch program, we identified 84 matches across 24 different cyclic peptides. This diversity of matches stems from the capability of single peptide templates to align variabily with multiple segments of the target protein, resulting in distinct binding scenarios that may involve the same or overlapping PDB entries. Among these, three peptides with PDB IDs 5EOC (13 residues), 2JRW (23 residues), and 2LWT (18 residues) were selected for further investigation based on rigorous visual inspection for effective steric matches and assessments of potential steric clashes with the oligosaccharide, as depicted in Fig. 1C. The cPEPmatch algorithm filters and superimposes cyclic peptides based on specified motifs and thresholds and automatically eliminates the matches that substantially overlap with the target’s interface. However, manual verification is still essential to ensure the biological relevance and structural accuracy of the computationally identified matches, especially given the unique and complex nature of protein-GAG interactions that the automated algorithm might not fully resolve. cPEPmatch is under continuous development, with future updates aiming to minimize the necessity for visual inspection by enhancing the algorithm’s precision and reducing manual steps. The selected matching peptides were chosen from the pool of cPEPs containing the most backbone residues matching the ATIII residues with significant contributions to the binding energy. Further optimization and refinement were then undertaken to improve their binding capabilities. When comparing Fig. 1C with Fig. 1B, it becomes evident that the two larger cyclic peptides, 2LWT and 2JRW, held the potential for additional mutations to incorporate more interacting residues vital for the ATIII binding site, as illustrated in Fig. 1C. Notably, as 2JRW lacks a well-defined secondary structure and exhibits considerable flexibility, one of its versions was enhanced with an internal cysteine bridge by mutating two specific residues to cysteine. Simulations, including the wild-type sequences of these two cyclic peptides, were also conducted as a baseline for comparison. The details of the mutated residues for all investigated cyclic peptides, including those matched with a consecutive motif, are comprehensively listed in Table 1.

#### 3.1.2 MD simulations and optimization

**Fig. 2:**
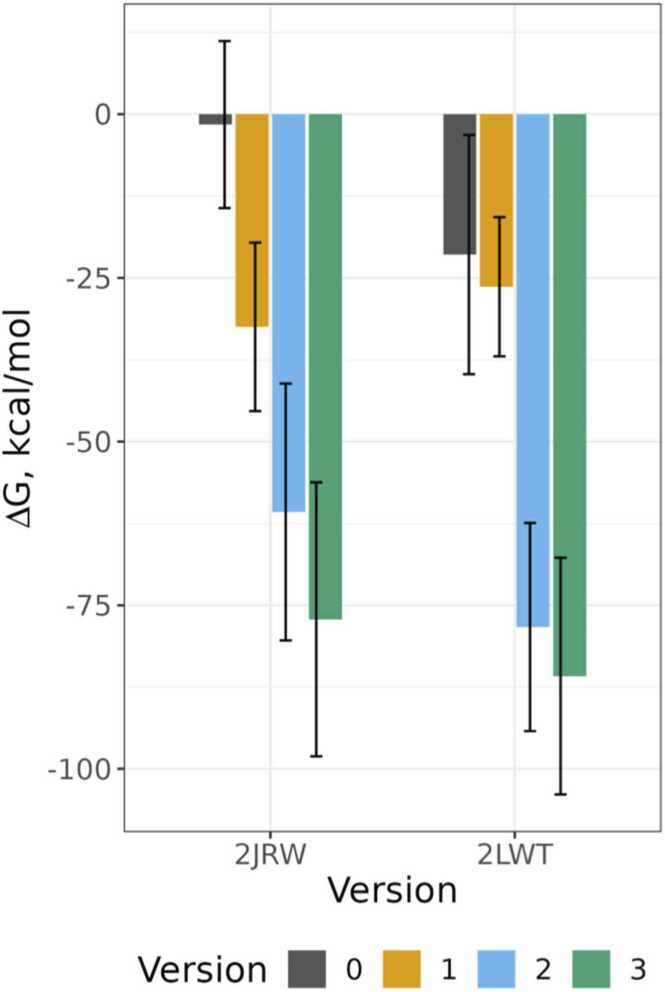
Δ*G* of all tested cPEP versions of 2JRW and 2LWT in complex with ligand 1, as calculated by MM-GBSA. Error bars represent the standard deviation over all frames from three 100 ns long independent runs.

**Fig. 3:**
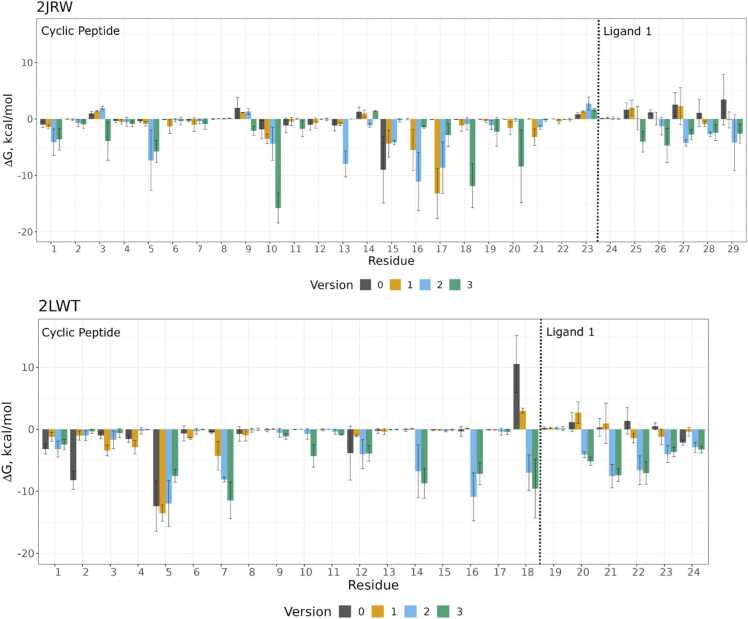
MM-GBSA per-residue ΔG decomposition for all tested cPEP versions of 2JRW and 2LWT in complex with ligand 1. Error bars indicate the standard deviation over all frames from three 300 ns runs.

We then analyzed the potential binding of the cyclic peptides to the GAG with MD simulations. The complexes of 2JRW and 2LWT, as derived from cPEPmatch and further manual refinements, were examined. Additionally, we included complexes with the wild-type sequences of these cyclic peptides as controls to monitor for nonspecific binding. MM-GBSA calculations were conducted for MD trajectories to determine the free energies of binding, following the same procedure as for the ATIII-ligand 1 complex. Fig. 2 shows the average total binding energy of all cyclic peptide-GAG complexes across the simulation trajectories, which suggests that the strongest binding was observed in the peptides where additional residues were incorporated after the visual inspection. The initial matches from cPEPmatch indicated only slight differences among the three cyclic peptides, yet all manifested stronger binding compared to their wild-type counterparts. Version 2 and 3 of 2LWT resulted in the most favorable free energies on average over three MD runs, although the differences with 2JRW were not significant. From the per-residue decomposed binding energies shown in Fig. 3, it is apparent that the enhanced binding affinities are due to the introduction of more ATIII-mimicking residues within the cyclic peptides. These residues are distributed across the peptide sequence, underscoring the significance of including sequentially non-consecutive residues in the binding site. The stability of these complexes was further supported by the RMSD analysis of both the GAG ligands and peptides, as presented in Fig. S1, with the 2LWT complexes demonstrating the highest stability during simulations.

#### 3.1.3 Umbrella sampling

**Fig. 4:**
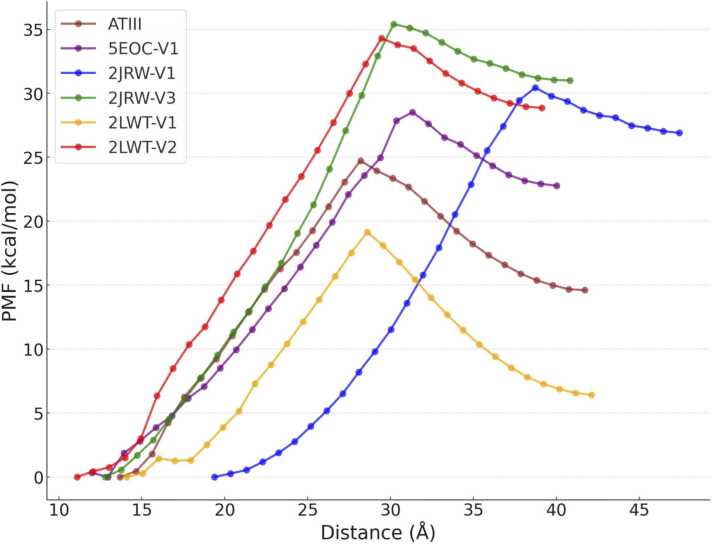
**:** Potential of mean force (PMF) plots for 2JRW (v1, v3), 2LWT (v1, v2), 5EOC (v1) and ATIII in complex with ligand 1. The applied force constant for the simulations was 4 kcal/mol/Å^2^.

While MD simulations confirmed stable and energetically favorable cPEP-GAG interactions besides 2JWR V0, our objective was to engineer binders with affinity similar or higher than that of ATIII. The considerable size disparity between ATIII (427 residues) and the cyclic peptides (13–23 residues) precludes direct comparison of binding free energies using MM-GBSA due to the difference in the number of interacting residues and the entropy contributions that are not accounted for in the smaller molecules. To facilitate a complementary way of comparison, umbrella sampling MD simulations were utilized, as proposed by Torrie and Valleau in 1977 [34], alongside the computation of potential mean force (PMF) plots from these distributions employing the weighted histogram analysis method (WHAM) with Grossfield's WHAM software [20], [8], as illustrated in Fig. 4. The PMF curves indicate that, relative to ATIII, the cPEP-match-derived versions of 2JRW and 5EOC exhibit stronger binding, while 2LWT version 1 demonstrates slightly weaker interaction. Notably, version 2 of 2LWT shows binding affinity comparable to the larger 2JRW variant. These results should be interpreted cautiously and qualitatively because despite the sufficient size of the periodic box used in these simulations, the observed decrease of the PMF curve after its maximum is potentially due to the polarization of the explicit solvent molecules in this charged system.

Interestingly, all cyclic peptide complexes except 2LWT V0 possess higher free energy barriers to dissociation compared to the ATIII complex. Despite their smaller size, the modified sequences of the peptides contain numerous positively charged residues that form strong electrostatic interactions with the GAG molecule. This observation may be attributed to the presence of residues in ATIII that are more critical for ligand 1 binding. Given its demonstrated strong binding affinity and stability in MD simulations, as shown in Figs. 2–4, alongside the consistent stability observed for this peptide in MD trajectories (Fig. S1), 2LWT version 2 was selected for further investigation into its binding specificity.

#### 3.1.4 Specificity of the ATIII-GAG interactions

Antithrombin III (ATIII) exhibits high specificity when binding the heparin mimetic as observed in the crystal structure (PDB ID: 1E03). To emulate a successful mimetic, our selected cyclic peptide must similarly exhibit specificity to the same GAG as ATIII. To assess this, we compared the peptide's binding to ligand 1 with its interaction with ligand 2, which differs from ligand 1 by a sulfated iduronic acid instead of glucuronic acid at position 2 and lacking the 3-*O* sulfate groups on glucosamine residues at positions 3 and 5, as illustrated in Fig. S3 A. Notably, the 3-*O* sulfate at position 5 is a key determinant for ATIII specificity [19], [33]. Furthermore, we also evaluated the effect of the ligand's initial binding pose. The first ligand starting pose, derived from cPEPmatch, aligns the cyclic peptide with the ATIII binding site from the ATIII-GAG experimental complex structure. For the second ligand starting pose, the GAG was slightly shifted from the corresponding experimental pose to allow for a different interaction profile with the cyclic peptide.

The binding free energies of 2LWT version 2 interacting with the different GAGs, calculated from the trajectories of three MD simulations for each ligand in both initial positions, are displayed in Fig. S3 B. The binding affinity for ligand 1 was more favorable compared to that for ligand 2, though the difference was statistically insignificant. This higher affinity could be explained by the presence of two additional sulfate groups in ligand 1. Interestingly, in the MD simulations with the second starting pose, ligand 1 moved back to a binding pose similar to the experimentally derived one, suggesting a more favorable interaction in the first starting pose. This specific binding pose alteration was not observed with ligand 2, implying a distinct specificity of the cyclic peptide for ligand 1. These findings indicate that the cyclic peptide exhibits certain binding specificity towards ligand 1 that is detected by our calculations.

The encouraging findings from our computational predictions, encompassing cPEPmatch, MM-GBSA, umbrella sampling, and specificity studies, motivated the experimental validation of the 2LWT version 2 cyclic peptide's binding. To this end, we proceeded to employ NMR and ITC techniques to assess the binding affinity of this peptide to the GAG ligand, aiming to validate the insights gained from our computational studies.

### 3.2 Experimental validation

#### 3.2.1 Solution NMR

Although cPEP 2LWT V2 contains a disulfide bridge, NMR measurements were conducted at 283 K to account for rapid NH exchange, consistent with observations for the wild-type 2LWT peptide [7]. Partial assignment of the peptide confirmed the disulfide bridge with a 99.4 % probability, according to POKY analysis.

Notably, mutations introduced in V2 shifted the secondary structure from an antiparallel beta-sheet described for V0 to a random coil, as illustrated in Fig. S2 and Table SI1. Upon ligand addition, negligible chemical shift perturbations were observed on Cα or Cβ. The most significant changes, less than 0.75 ppm in the ^13^C chemical shift, were associated with one Alanine and one Cysteine residue. This suggests that ligand binding does not significantly affect the peptide’s secondary structure.

Using the ^1^H–^1^H TOCSY, assignments could be transferred to spectra with 100 µM cPEP 2LWT V2 at pH 7 used for ligand binding.

**Fig. 5:**
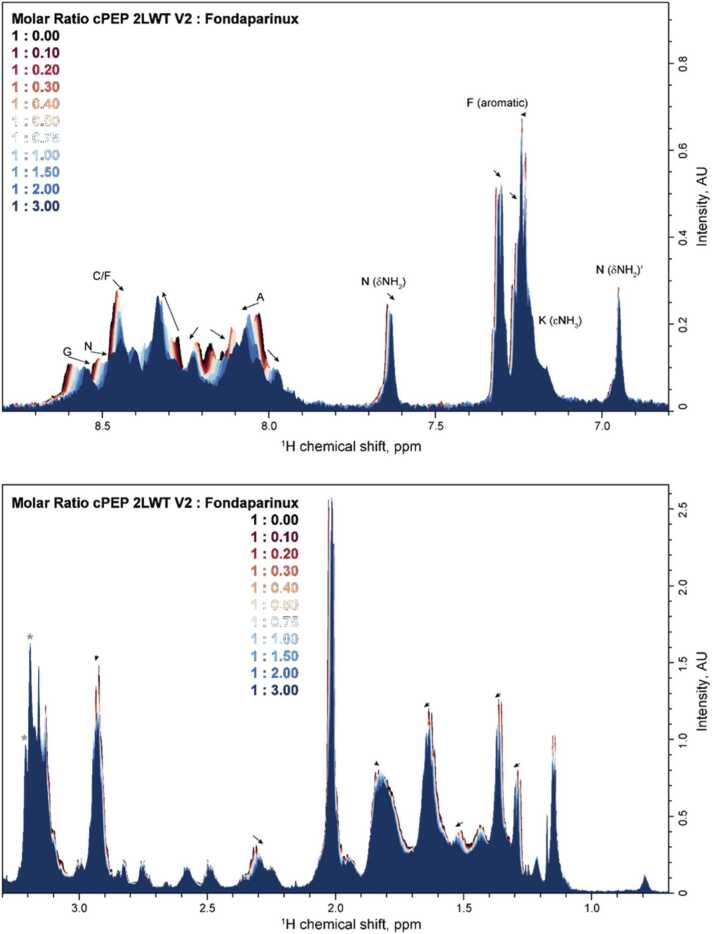
^1^H NMR spectra at different ratios of cPEP 2LWT V2 and Fondaparinux from black (0) to red and blue (3 fold excess of Fondaparinux). The upper panel shows the amide and aromatic region, while the lower panel shows the aliphatic region. Arrows correspond to the shift direction upon addition of the ligand, while the grey star indicates signals from the ligand Fondaparinux. Where the amino acid could be identified, which is responsible for the shift indicated by the arrow, the arrow is labeled.

In the 1D ^1^H spectra with increasing concentration of Fondaparinux that differs from ligand 1 by an additional methyl group at the reducing end, the dNH_2_ signals of the N3 could be easily identified (7.65 and 6.95 ppm). In between, the aromatic signals of F11 resonate, but also the εNH_3_ from the three Lysines appear as shoulder. Upon addition of the ligand Fondaparinux, the backbone NH signals showed the highest alterations in their chemical shift (Fig. 5, upper panel). While both Glycines (G1 and G9) showed a remarkable upfield shift, only the downfield Cysteine (C6 or C15) was highly influenced by the addition of the ligand. The upfield cysteine showed only a slight perturbation (smaller than 0.01 ppm) of the chemical shift. While nearly all assigned amino acids showed influences of their NH resonance with the addition of the ligand, the methionine resonances (M10 and M17, overlapping) are also undisturbed. The sidechain NHs and aromatic signals showed only minor or no influences as well as the aliphatic signals (Fig. 5, lower panel). Taken together, while the alterations of the chemical shift of the cyclic peptide 2LWT V2 during the course of titration clearly demonstrate the binding of this cyclic peptide to Fondaparinux, the observed data does not allow an elucidation of the binding site.

**Fig. 6:**
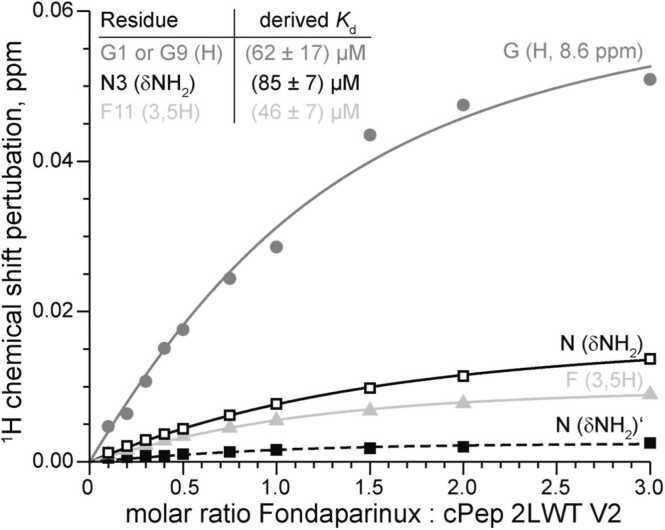
^1^H chemical shift perturbations with increasing amount of Fondaparinux on resolved residues of 2LWT2 V2 with derived *K*_*d*_ ± standard deviation. The dashed line for N (δNH_2_)’ is a guidance for the eye; no *K*_*d*_ was calculated as shifts are too small.

Despite the high overlap in the 1D proton spectrum of the 18 amino acids, four resolved signals over the course of titration were used for further analysis. Namely the both δNH_2_ (δNH_2_ and δNH_2_’) from N3, the backbone H resonance from the downfield Glycine (G1 or G9) and the aromatic protons (3,5) of the F11 sidechain (see labels in Fig. 5). The perturbations of the chemical shift (CSP) upon addition of Fondaparinux are shown in Fig. 6. From that, except for dNH_2_’ of N3 (dashed line), dissociation constants were calculated. We calculated an averaged *K*_*d*_ of (64 ± 16) µM from the three derived *K*_*d*_s.

#### 3.2.2 ITC assessment

**Fig. 7:**
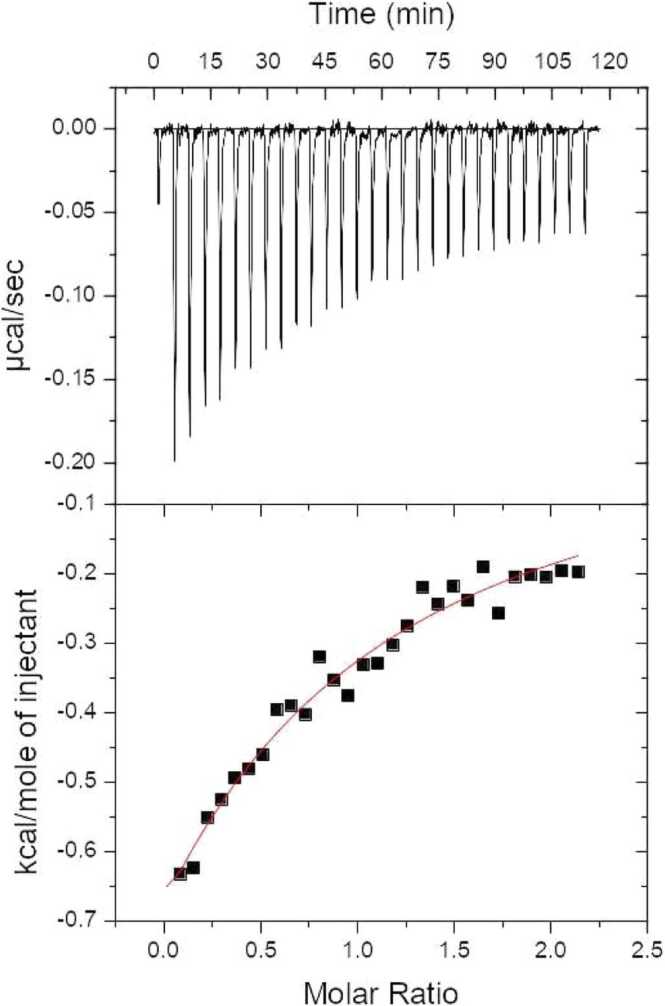
Calorimetric titration isotherms of the binding interactions between cPEP and Fondaparinux in 20 mM NaPi buffer (150 mM NaCl, pH 7.0), at 298.15 K.

The interaction between cPEP and Fondaparinux was quantitatively assessed using Isothermal Titration Calorimetry (ITC) (Fig. 7). Thermodynamic parameters were directly obtained from ITC measurements and included log K_ITC_ = 4.15 ± 0.15 and ∆H_ITC_ = −0.86 ± 0.11 kcal/mol. The corresponding *K*_*d*_ is 73.2 ± 26.8 µM, which is slightly higher than the *K*_*d*_ obtained by NMR. This can be explained from ITC experiments conducted at 15 degrees higher temperature. Data fitting was carried out using nonlinear least-squares procedures, based on a model assuming a single set of equivalent binding sites. The stoichiometry for the interaction, set at a 1:1 ratio of peptide to oligosaccharide, was held constant for the best fit to experimental data. Additional thermodynamic parameters, namely the free energy of binding ∆G_ITC_ = −5.72 ± 0.20 kcal/mol and entropy change T ∆S_ITC_ = 4.9 kcal/mol, were calculated based on standard relationships:

∆G_ITC_ = −RT ln K_ITC_ = ∆H_ITC_ − T ∆S_ITC_

ITC data indicate that the formation of a stable cPEP-Fondaparinux complex is primarily entropy-driven (|∆H_ITC_| < |T∆S_ITC_|). However, a modest contribution from enthalpy change to the free energy of binding was also observed. Therefore, the entropic component - potentially originating from the solvent restructuration upon binding - is crucial for the complex formation.

## 4 Conclusion

Our study has made a stride in the computational analysis of GAG-binding cyclic peptides, showcasing the potential of advanced computational tools in the discovery and optimization of novel peptide-based therapeutics. By adapting the cPEPmatch algorithm to effectively identify and characterize complex binding sites typical of protein-GAG interactions, we have laid a foundation for the systematic exploration of cyclic peptides as GAG binders. The successful identification of lead peptide binders, especially 2LWT version 2, whose binding was supported by MD analysis as well as by NMR and ITC experiments, highlights the efficacy of our computational approach. The most favorable calculated binding affinities of the designed peptides are still, however, weaker than those of the wild-type ATIII-ligand 1 complex (−111 kcal/mol) [30]. Experimentally, the affinity between ATIII and ligand 1 was obtained in the nanomolar range which is essentially higher than the ones for the designed peptides [2], [25]. Nevertheless, this proof-of-principle study not only advances our understanding of peptide-GAG interactions but also opens avenues for future research and development in the field of cyclic peptide therapeutics, providing a strong starting point for further optimization of these peptide ligands. The methodologies and findings from this study can significantly aid in the discovery and optimization of new GAG-binding cyclic peptides, paving the way for potential therapeutic applications.

## CRediT authorship contribution statement

**Sven Rothemund:** Validation, Methodology, Investigation. **Dariusz Wyrzykowski:** Validation, Methodology, Investigation. **Martin Zacharias:** Writing – review & editing, Supervision, Funding acquisition, Conceptualization. **Anja Penk:** Writing – review & editing, Validation, Methodology, Investigation. **Margrethe Gaardløs:** Writing – original draft, Visualization, Validation, Investigation, Formal analysis, Data curation. **Brianda L Santini:** Writing – review & editing, Writing – original draft, Visualization, Software, Methodology, Investigation, Formal analysis, Data curation, Conceptualization. **Sergey Samsonov:** Writing – review & editing, Supervision, Funding acquisition, Conceptualization.

## Declaration of Competing Interest

The authors declare that they have no known competing financial interests or personal relationships that could have appeared to influence the work reported in this paper.
