# Supplementary material for: Rational design of glycosaminoglycan binding cyclic peptides using cPEPmatch
Source: Comput Struct Biotechnol J. 2024 Jul 20;23:2985–94. doi: 10.1016/j.csbj.2024.07.016 (PMC11318538; doi:10.1016/j.csbj.2024.07.016)
Supplement: Figure S1 — Supplementary material [file mmc1.docx]

# **Supplementary Material**


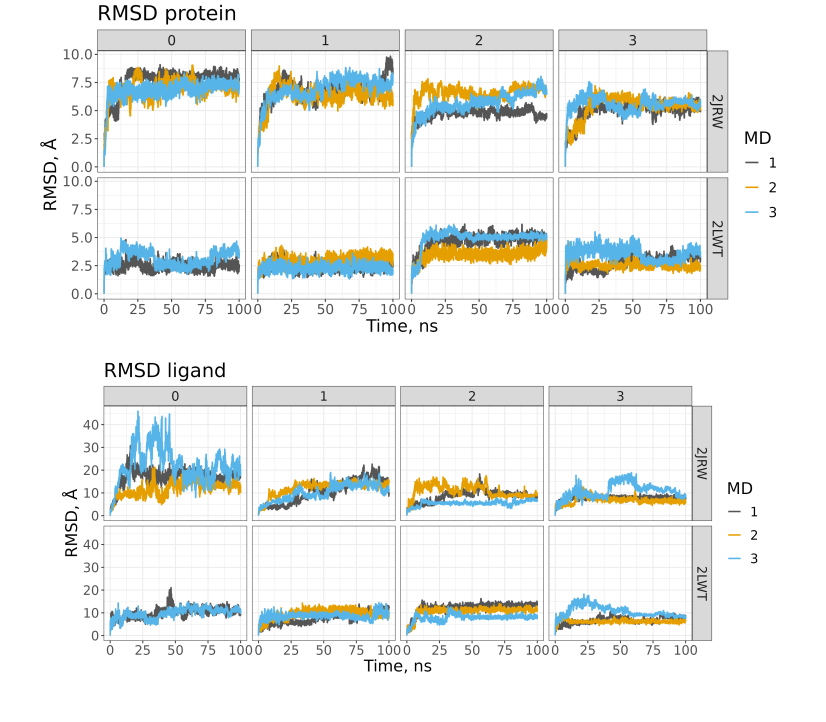


Figure S1: RMSD plots for the ligand 1 and protein over the entire simulation trajectory. The data are shown for cPEP matches 2JRW and 2LWT, illustrating the stability of these complexes over time.


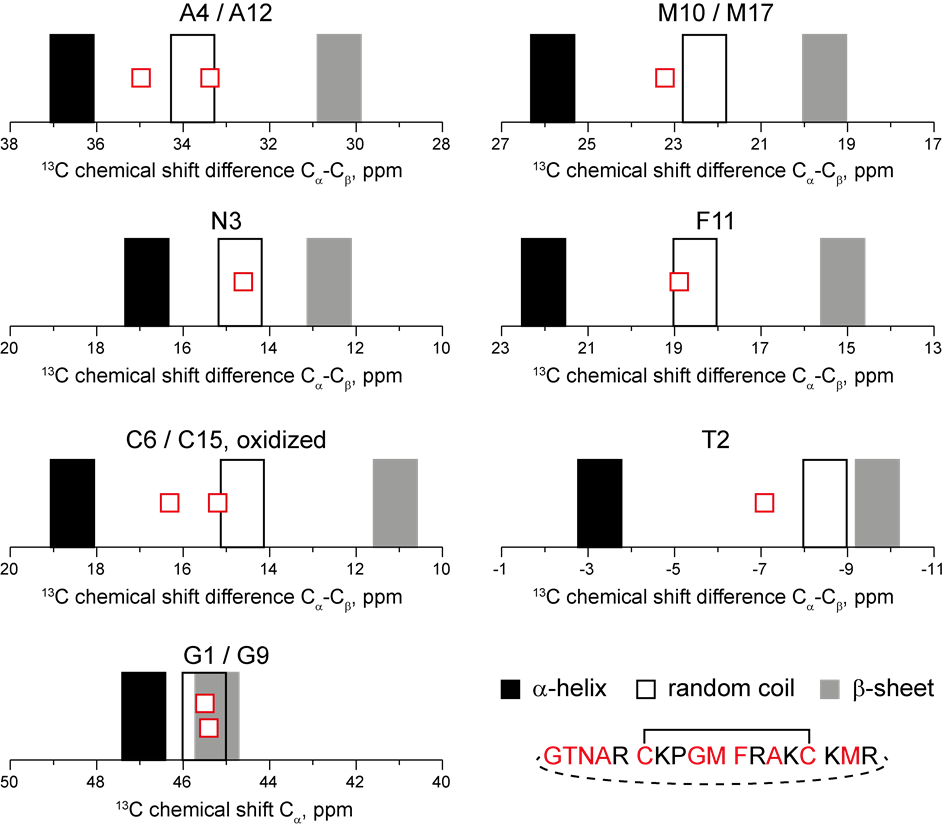


Figure S2: Calculated chemical shift differences between C_⍺_ and C_β_ (red boxes) for each assigned amino acid type in cPEP 2LWT V2. For Glycine (G1/G9) only the C_⍺_ shift is given. The difference between C_⍺_ and C_β_ is dependent on the secondary structure. The standard differences for each amino acid according to the respective secondary structure element are given as bars for reference. A chemical shift difference near the black bar represents a standard value for the respective amino acid type if it is in a ⍺-helical secondary structure element. White and grey bars are indicative for random coil and β-sheet structure elements, respectively. Arginines, Lysines and Proline were not assigned and are labeled black in the sequence given in the right lower corner. The red labeled, assigned amino acids cover 61% of the peptide sequence and are all in the range of for a random coil structure. Hence, the introduced mutations in cPEP 2LWT V2 lead to a random coil structure, while for the original peptide 2LWT V0 an antiparallel beta sheet structure was reported [(Elliott *et al.*, 2014)](https://www.zotero.org/google-docs/?zppkIb).

**
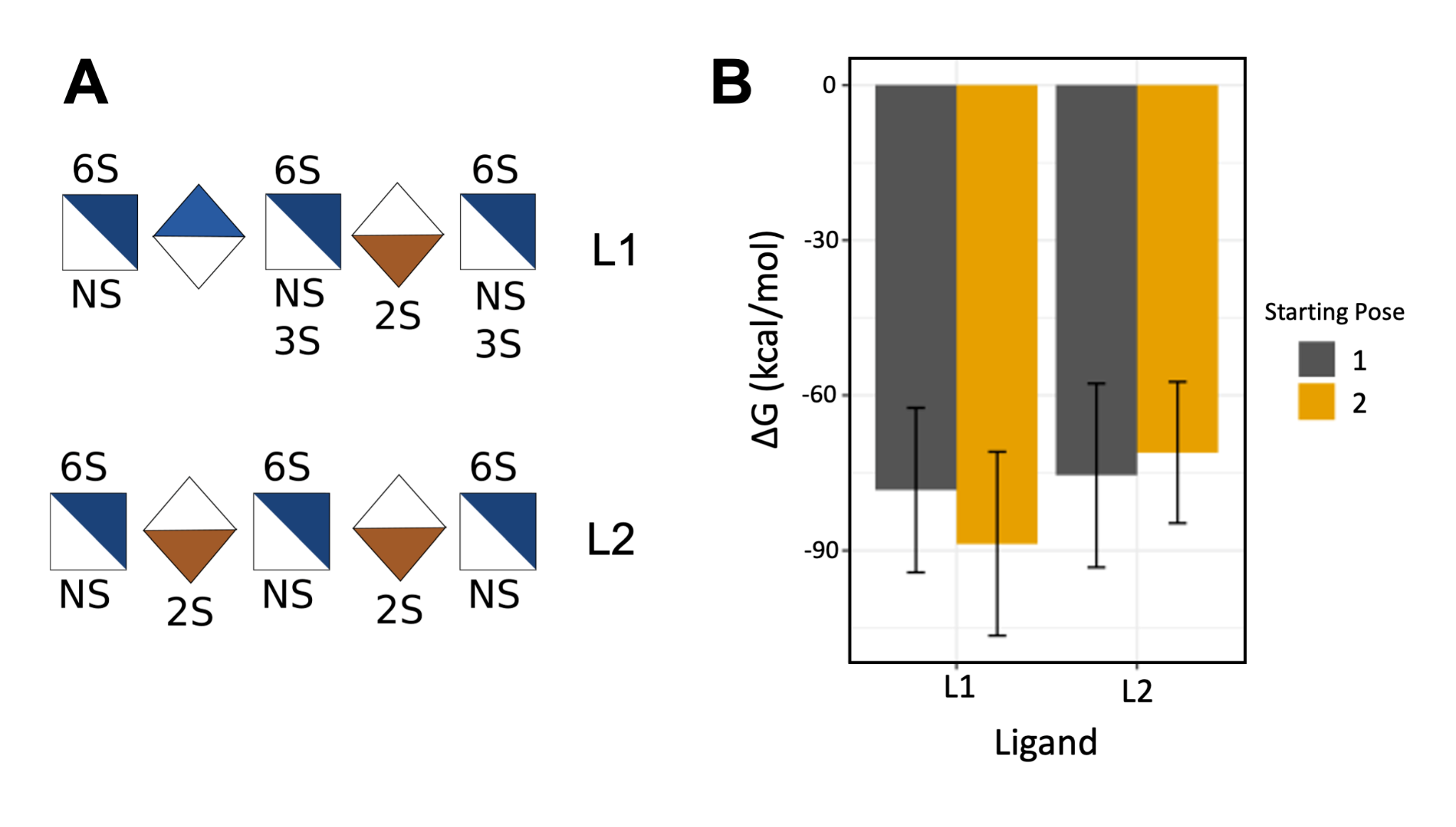
**

Figure S3: A) Structures of ligands 1 and 2. B) ΔG values from MM-GBSA based on three MD simulations of the 2LWT V2 cyclic peptide with ligands 1 (L1) and 2 (L2), each starting from two distinct starting poses (1 and 2).

Table S1.**:** Chemical shifts for the assigned classes of amino acids at pH 6.4 and 283K with their respective standard deviation.

| **Class** | **Residue** | **group** | **Shift in ppm** | **SD in ppm** |
| --- | --- | --- | --- | --- |
| Ala (A4 / A14) | a | C⍺ | 52.98 | 0.03 |
|  |  | Cꞵ | 19.60 | 0.04 |
|  |  | NH | 8.047 | 0.003 |
|  |  | H⍺ | 4.187 | 0.005 |
|  |  | Hꞵ | 1.287 | 0.005 |
|  | b | C⍺ | 54.05 | 0.04 |
|  |  | Cꞵ | 19.08 | 0.01 |
|  |  | NH | 8.281 | 0.001 |
|  |  | H⍺ | 4.104 | 0.007 |
|  |  | Hꞵ | 1.361 | 0.007 |
| Asn (N3) | N3 | C⍺ | 53.49 | 0.06 |
|  |  | Cꞵ | 38.88 | 0.07 |
|  |  | NH | 8.534 | 0.002 |
|  |  | H⍺ | 4.655 | 0.005 |
|  |  | Hꞵ | 2.829 | 0.004 |
|  |  | Hꞵ' | 2.758 | 0.006 |
| Cys (C6 / CC15) | a | C⍺ | 56.73 | 0.06 |
|  |  | Cꞵ | 41.53 | 0.01 |
|  |  | NH | 8.128 | 0.001 |
|  |  | H⍺ | 4.470 | 0.006 |
|  |  | Hꞵ | 3.160 | 0.005 |
|  |  | Hꞵ' | 3.088 | 0.005 |
|  | b | C⍺ | 55.33 | 0.04 |
|  |  | Cꞵ | 39.02 | 0.11 |
|  |  | NH | 8.474 | 0.002 |
|  |  | H⍺ | 4.649 | 0.008 |
|  |  | Hꞵ | 3.118 | 0.004 |
|  |  | Hꞵ' | 2.911 | 0.003 |
| Gly (G1 / G9) | a | C⍺ | 45.32 | 0.06 |
|  | (downfield) | NH | 8.619 | 0.001 |
|  |  | H⍺ | 4.097 | 0.008 |
|  |  | H⍺' | 3.708 | 0.011 |
|  | b | C⍺ | 45.50 | 0.04 |
|  | (upfield) | NH | 8.374 | 0.005 |
|  |  | H⍺ | 4.119 | 0.008 |
|  |  | H⍺' | 3.874 | 0.027 |
| Met (M10 / M17) | overlap of | C⍺ | 56.07 | 0.05 |
|  | residues | Cꞵ | 32.85 | 0.09 |
|  |  | Cγ | 32.27 | 0.04 |
|  |  | Cε | 17.07 | 0.03 |
|  |  | NH | 8.113 | 0.144 |
|  |  | H⍺ | 4.391 | 0.005 |
|  |  | Hꞵ | 2.068 | 0.010 |
|  |  | Hꞵ' | 1.987 | 0.009 |
|  |  | Hγ | 2.587 | 0.007 |
|  |  | Hγ' | 2.492 | 0.006 |
|  |  | Hε | 2.013 | 0.010 |
| Phe (F11) | F11 | C⍺ | 57.94 | 0.03 |
|  |  | Cꞵ | 39.05 | 0.03 |
|  |  | NH | 8.484 | 0.001 |
|  |  | H⍺ | 4.602 | 0.008 |
|  |  | Hꞵ | 2.993 | 0.005 |
|  |  | Hꞵ' | 3.175 | 0.010 |
| Thr (T2) | T2 | C⍺ | 62.62 | 0.04 |
|  |  | Cꞵ | 69.70 | 0.04 |
|  |  | Cγ | 21.75 | 0.04 |
|  |  | NH | 8.230 | 0.006 |
|  |  | H⍺ | 4.244 | 0.005 |
|  |  | Hꞵ | 4.224 | 0.007 |
|  |  | Hγ | 1.154 | 0.005 |

### **Abbreviations**

• DIEA: N,N-diisopropylethylethylamine

• DMF: N,N-Dimethylformamide

• HCTU: O-(1H-6-Chlorobenzotriazole-1-yl)-1,1,3,3-tetramethyluronium hexafluorophosphate

• HOBT: 1-Hydroxy-1H-benzotriazole hydrate

• NMM: 4-Methylmorpholine

• PyBOP: Benzotriazole-1-yl-oxy-tris-pyrrolidino-phosphonium hexafluorophosphate

• TFA: Trifluoroacetic acid

• TMSP-d_4:_ 3-(Trimethylsilyl)propionic-2,2,3,3-d₄ acid
